# Detection of chromosomal aneuploidy in ancient genomes

**DOI:** 10.1038/s42003-023-05642-z

**Published:** 2024-01-11

**Authors:** Kyriaki Anastasiadou, Marina Silva, Thomas Booth, Leo Speidel, Tony Audsley, Christopher Barrington, Jo Buckberry, Diana Fernandes, Ben Ford, Mark Gibson, Alexandre Gilardet, Isabelle Glocke, Katie Keefe, Monica Kelly, Mackenzie Masters, Jesse McCabe, Lauren McIntyre, Paola Ponce, Stephen Rowland, Jordi Ruiz Ventura, Pooja Swali, Frankie Tait, David Walker, Helen Webb, Mia Williams, Annsofie Witkin, Malin Holst, Louise Loe, Ian Armit, Rick Schulting, Pontus Skoglund

**Affiliations:** 1 Ancient genomics laboratory, The Francis Crick Institute, London, United Kingdom; 2 Genetics Institute, University College London, London, United Kingdom; 3 Independent Scholar, Wells, United Kingdom; 4 Bioinformatics and Biostatistics Science Technology Platform, The Francis Crick Institute, London, United Kingdom; 5 School of Archaeological and Forensic Sciences, University of Bradford, Bradford, United Kingdom; 6 Network Archaeology, Lincoln, United Kingdom; 7 Oxford Archaeology, Oxford, United Kingdom; 8 York Osteoarchaeology, York, United Kingdom; 9 On-Site Archaeology, York, United Kingdom; 10 Department of Archaeology, University of York, York, United Kingdom; 11 Wells and Mendip Museum, Wells, United Kingdom; 12 School of Archaeology, University of Oxford, Oxford, United Kingdom

**Keywords:** Population genetics, Evolutionary genetics

## Abstract

Ancient DNA is a valuable tool for investigating genetic and evolutionary history that can also provide detailed profiles of the lives of ancient individuals. In this study, we develop a generalised computational approach to detect aneuploidies (atypical autosomal and sex chromosome karyotypes) in the ancient genetic record and distinguish such karyotypes from contamination. We confirm that aneuploidies can be detected even in low-coverage genomes ( ~ 0.0001-fold), common in ancient DNA. We apply this method to ancient skeletal remains from Britain to document the first instance of mosaic Turner syndrome (45,X0/46,XX) in the ancient genetic record in an Iron Age individual sequenced to average 9-fold coverage, the earliest known incidence of an individual with a 47,XYY karyotype from the Early Medieval period, as well as individuals with Klinefelter (47,XXY) and Down syndrome (47,XY, + 21). Overall, our approach provides an accessible and automated framework allowing for the detection of individuals with aneuploidies, which extends previous binary approaches. This tool can facilitate the interpretation of burial context and living conditions, as well as elucidate past perceptions of biological sex and people with diverse biological traits.

## Abstract (web summary)

A sex chromosome identification method for ancient DNA revealed six individuals who lived with chromosomal aneuploidies in Britain from ~ 2,500 to 250 years ago. Genomic and osteological information was combined to shed light on their lives.

## Introduction

The study of ancient genomes has revolutionised our ability to examine human biology over thousands of years, providing insight into phenotypic variation, social stratification and their impact on health throughout history^1,2^. In particular, ancient DNA can contribute to our understanding of chromosomal sex in the past, its relationship with gender, and allow for the identification of non-binary chromosomal sex via investigation of the number of sex chromosomes detected in an individual’s karyotype. Deploying genomic data for sex identification can surpass limitations in the sex estimation methods that rely on osteological features, as those can be less accurate or inapplicable when human skeletons are only partially complete^3–5^. Moreover, the subtlety of prepubescent sexual dimorphism means that established osteological sex estimates are rarely applicable to nonadult remains^6,7^. Several methods have been used to identify chromosomal sex from low-coverage genomes by comparing the proportion of sequences aligning to chromosome Y with sequences aligning to chromosome X or to autosomes^8–10^. However, publicly available tools do not aim to detect other, less frequent, combinations of sex chromosomes or aneuploidies.

Embryonic aneuploidies vary in their severity from pregnancy loss to milder conditions, like Klinefelter syndrome^11^. Klinefelter syndrome is characterised by infertility and a slightly increased risk for disorders like type 2 diabetes. The majority of individuals with Klinefelter syndrome carry a 47,XXY karyotype, accounting for 1 in 500–650 newborn males, which makes it the most common chromosomal disorder in males^12^. Although other karyotypes with supernumerary X chromosomes have been described (e.g. 48,XXXY or 49,XXXXY), these occur at much lower frequencies^13^. Additional copies of chromosome Y (47,XYY), observed in about 1 in 1000 male births, are associated with taller than average stature and an increased risk for asthma, seizures and learning disabilities^14,15^. On the other hand, individuals with Turner syndrome, a condition that affects 1 in 2500–3000 live female births^16^, carry only a single chromosome X with karyotype 45,X0 or in mosaic form (e.g. 45,X0/46,XX or 45X0/46,XY). Somatic presentation of Turner syndrome depends on the presence or degree of mosaicism and is associated with shorter stature and a higher incidence of infertility, cardiovascular, renal and endocrine disease^17^. Autosomal aneuploidies that survive to birth are trisomies 13, 18 and 21 and they are often associated with developmental and heart issues, with trisomy 21 (Down syndrome) being the most common one by far^18^.

Klinefelter is the only aneuploidy observed multiple times in the ancient genomic record to date, with reports of individuals with a 47,XXY karyotype in Copper Age Iberia^19^, Early Medieval Finland^20^, Viking Age Orkney^21^ and Medieval Portugal^22^ and Iceland^23^. Other aneuploidies identified with ancient DNA include a neonate with a 47,XXX karyotype (trisomy X, or triple X syndrome) from Iberia from the second millennium BCE^19^ and a neonate from Neolithic Ireland with trisomy 21^24^. The ability to detect these atypical karyotypes in the ancient genomic record has the dual advantage of providing a more detailed insight into the life conditions and societal surroundings of those individuals, while also adding a historical perspective for medical practitioners and patients today.

In this paper, we develop and validate a method that distinguishes between all sex chromosome karyotypes, as well as detecting autosomal aneuploidies such as trisomy 21. We apply this method to new data from ancient Britain and present the oldest known instance of mosaic Turner syndrome dating to the Early Iron Age, three individuals with Klinefelter syndrome, spanning from the Iron Age to the Post-Medieval Period from England, an individual with 47,XYY syndrome in Early Medieval England and an Iron Age infant with Down syndrome.

## Results

### A computational method to identify aneuploidies using ancient DNA

Our computational approach is based on independently quantifying the number of observed sequences aligning to any chromosome compared to an ‘autosomal baseline’, *N*_a_, which we defined as the sum of sequences aligning to chromosomes 1 through 22, excluding chromosomes 13, 18 and 21 which are the only autosomal aneuploidies that survive to birth in appreciable numbers^25^. We then defined the following metrics. The *R*_x_ estimate was calculated by dividing the number of sequences aligning to chromosome X with *N*_a_, and the *R*_y_ estimate was calculated by dividing the number of sequences aligning to chromosome Y with *N*_a_. We estimated the theoretical values for *N*_a_, *R*_x_, and *R*_y_ expected for chromosome sizes in human genome build 37 and applied our method to published shotgun-sequenced genomes to investigate their overlap with the theoretical values. Leveraging the observed distribution of the empirical data, we optimised the assignment thresholds used to estimate the number of copies of chromosomes X and Y in the karyotype. Additionally, we defined an alternative set of assignment thresholds, optimised for use with target enriched libraries on the widely used “1240k” SNP positions^26,27^ (see Supplementary Note 4 and Supplementary Figure 3). We obtained standard errors for the assignments using binomial approximations, and used these to test if individuals are significantly deviating from theoretical expectations.

**Fig. 1:**
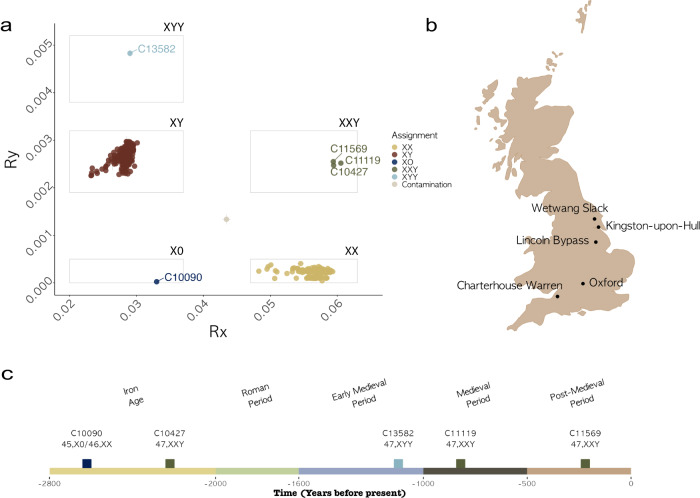
Five individuals with sex chromosomal aneuploidies detected using ancient DNA. **a** *R*_x_ and *R*_y_ estimates, representing the number of sequences aligning to chromosomes X and Y, respectively, as a proportion of the sum of sequences aligned to autosomes, for 134 genomes from Antonio et al.^28^, 436 genomes from Margaryan et al^21^. and 5 newly published individuals with sex chromosomal aneuploidies (*n* = 575) (Supplementary Data 2). **b** Map of Great Britain with the location of the five sites of origin of individuals presented in this study. **c** Timeline with archaeological periods of British history and prehistory, spanning from the Iron Age to the present, including dates and karyotypes of newly published individuals with sex chromosomal aneuploidies.

The method described here was applied to 570 published ancient shotgun sequenced genomes from Viking Age Northern Europe^21^ and ancient Rome^28^, representing two independent studies from different laboratories to corroborate the absence of batch effects. Individuals with a 46,XX karyotype clustered close to the mean expected *R*_x_ value of 0.056 for two copies of chromosome X and their *R*_y_ values were close to 0. Meanwhile, individuals with a 46,XY karyotype had *R*_x_ values within the typical range expected for a single copy of chromosome X (0.028), while having *R*_y_ values close to the mean value of 0.0026, expected of individuals carrying one chromosome Y (Fig. 1a).

### Detection of individuals with 47,XXY and 47,XYY karyotypes

**Fig. 2:**
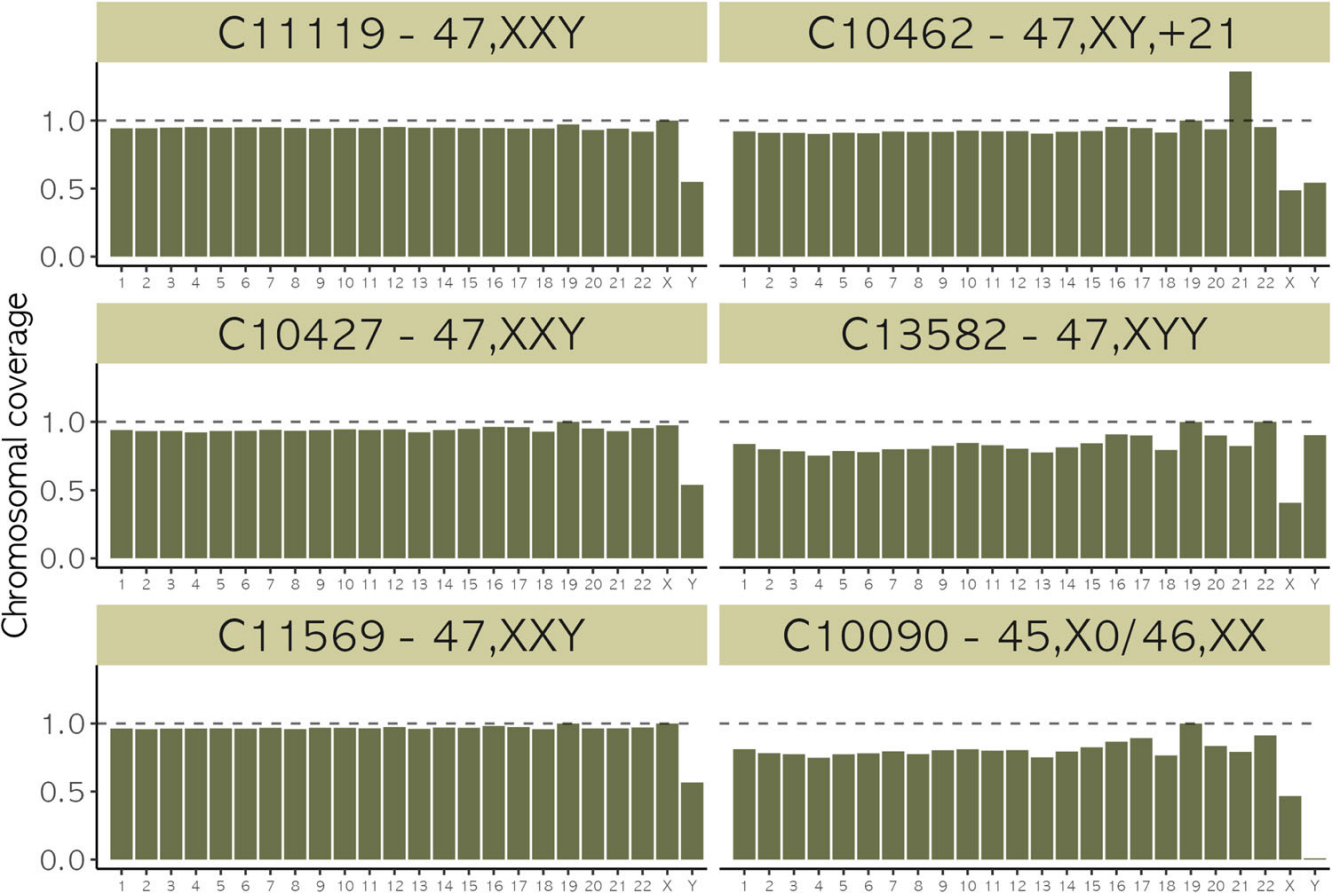
Per chromosome coverage in five individuals with aneuploidies on the sex chromosomes and in one individual with aneuploidy on chromosome 21 from this study. Proportion of sequences aligned on each chromosome, normalised between chromosomes and across individuals to account for differences in sequencing effort per library (Supplementary Data 3). Individuals C11119 (Sk4205 from Longwall Quad, Magdalen College), C10427 (WS224 from Wetwang Slack), C11569 (Sk30828 from Trinity Burial Ground) with a 47,XXY karyotype carried two copies of chromosome X as well as one copy of chromosome Y. Individual C10462 (WS267 from Wetwang Slack) carried a typical number of sex chromosomes (one X and one Y) and an extra copy of chromosome 21 resulting in a 47,XY, + 21 karyotype. Individual C13582 (Skeleton 16586 from Lincoln Eastern Bypass) had a 47,XYY karyotype, as indicated by the presence of only one copy of chromosome X and two copies of chromosome Y. Individual C10090 (CH163 from Charterhouse Warren) carried no copies of chromosome Y and one copy of chromosome X, with mosaicism (i.e. a minority of cells carried a 46,XX karyotype as evidenced by heterozygosity in chromosome X).

Three individuals, published in this study, clustered in the top right corner in Fig. 1a, having an *R*_x_ value within the typical range observed in XX individuals and an *R*_y_ value within the typical range for XY individuals. This indicated the presence of two copies of chromosome X and one copy of chromosome Y and suggested that these three individuals (C10427, C11119, C11569) had Klinefelter syndrome (47,XXY). Details about their age and site of origin can be found in Table 1. Contamination estimates based on heterozygosity on chromosome X, commonly used to measure contamination in males, were high (>20%) for C11119 (Sk4205 from the medieval cemetery under Longwall Quad, Magdalen College) and C11569 (Sk30828 from Trinity Burial Ground), consistent with the presence of more than one X chromosome^29^ (Supplementary Data 1). However, C10427 (WS224 from Wetwang Slack) had a contamination estimate in the typical range of XY individuals (i.e., carrying only one X chromosome). Lack of heterozygosity is a strong indication that the supernumerary chromosome resulted from a copy of the maternal X occurring due to a mitotic error at an early postzygotic stage^30^. Individual C13582 had the highest observed *R*_y_ value out of all studied individuals (*R*_y_ = 0.0048) suggesting the presence of an additional copy of Y chromosome in their karyotype, which was also evident using the coverage per chromosome results (Fig. 2).

**Table 1:** Details of the studied individuals carrying chromosomal aneuploidies.

Sample ID	Skeleton ID	Human skeletal element	Archaeological site	Absolute/relative dates	Karyotype	Age at death
C10090	CH163	Right mandibular second molar	Charterhouse Warren	Iron Age (2703–2365 cal BP, cal AD 754–416)	45,X0/46,XX	18–22 y
C10427	WS224 (Gen Lab 199)	Left stapes	Wetwang Slack	Iron Age	47,XXY	18–19 y
C10462	WS267 (Gen Lab 162)	Right temporal	Wetwang Slack	Iron Age	47,XY, + 21	Neonate
C11119	Sk4205	Left incus	Medieval cemetery under Longwall Quad, Magdalen College, Oxford	Medieval Period (cal AD 1050–1290)	47,XXY	36–45 y / 45+ y
C11569	Sk30828	Right temporal	Trinity Burial Ground, Kingston-Upon-Hull	Post-medieval Period (early 19th c.)	47,XXY	16–19 y
C13582	16586	Right malleus	Lincoln Eastern Bypass	Early Medieval Period (8th c.)	47,XYY	46+ y

Information on radiocarbon dating and associated quality control data for absolute dates can be found in Supplementary Table 1 (C10090, Skeleton ID CH163) and Supplementary Table 2 (C11119, Skeleton ID 4205).

### Detection of an Iron Age individual with mosaic Turner syndrome (45,X0/46,XX)

Individual C10090 (CH163 from Charterhouse Warren) had an *R*_x_ value (0.033), indicating the presence of one copy of chromosome X and an *R*_y_ value (2.32 × 10^−5^) suggesting the absence of any copies of chromosome Y, thus revealing a 45,X0 karyotype, characteristic of Turner syndrome. Deeper sequencing (final average coverage 9.8x), confirmed the 45,X0 karyotype (Fig. 2).

However, upon closer investigation of the genome of C10090, two observations were suggestive of mosaicism. First, the estimated *R*_x_ value was higher than the *R*_*x*_ values of 375 tested individuals identified as XY (5.1 SD from the mean *R*_x_ for XY) (Fig. 1a), suggesting the presence of additional chromosome X sequences. Second, heterozygosity estimates^29^ on polymorphic sites of chromosome X were closer to those of individuals with two copies of chromosome X (16.37%), even when restricting the analysis to authentic ancient DNA sequences to exclude contamination^31^ (Supplementary Data 1). This does not seem related to structural variation, like the presence of an isochromosome, duplications or deletions of whole regions, that could have explained the observed additional X chromosome sequences, since there was even coverage and consistent levels of heterozygosity across the short and long arms of the chromosome (Supplementary Note 2 and Supplementary Fig. 1).

Hence, an interpretation accommodating a 45,X0 karyotype with a small but substantial excess of chromosome X sequences was mosaicism (45,X0/46,XX) i.e., a smaller proportion of the cells of C10090 carrying a typical 46,XX and the majority of cells carrying a 45,X0 karyotype. Indeed, the majority of adults with Turner syndrome exhibit some degree of mosaicism^32,33^. Mosaic presentations have also been detected in large-scale genomic datasets like the UK Biobank, where the 45,X0/46,XX karyotype was 6 times more prevalent than the non-mosaic 45,X0 and it was associated with more average stature and fewer reproductive and cardiovascular complications^34,35^.

### Statistical power to detect aneuploidy at low genomic sequencing coverages

**Fig. 3:**
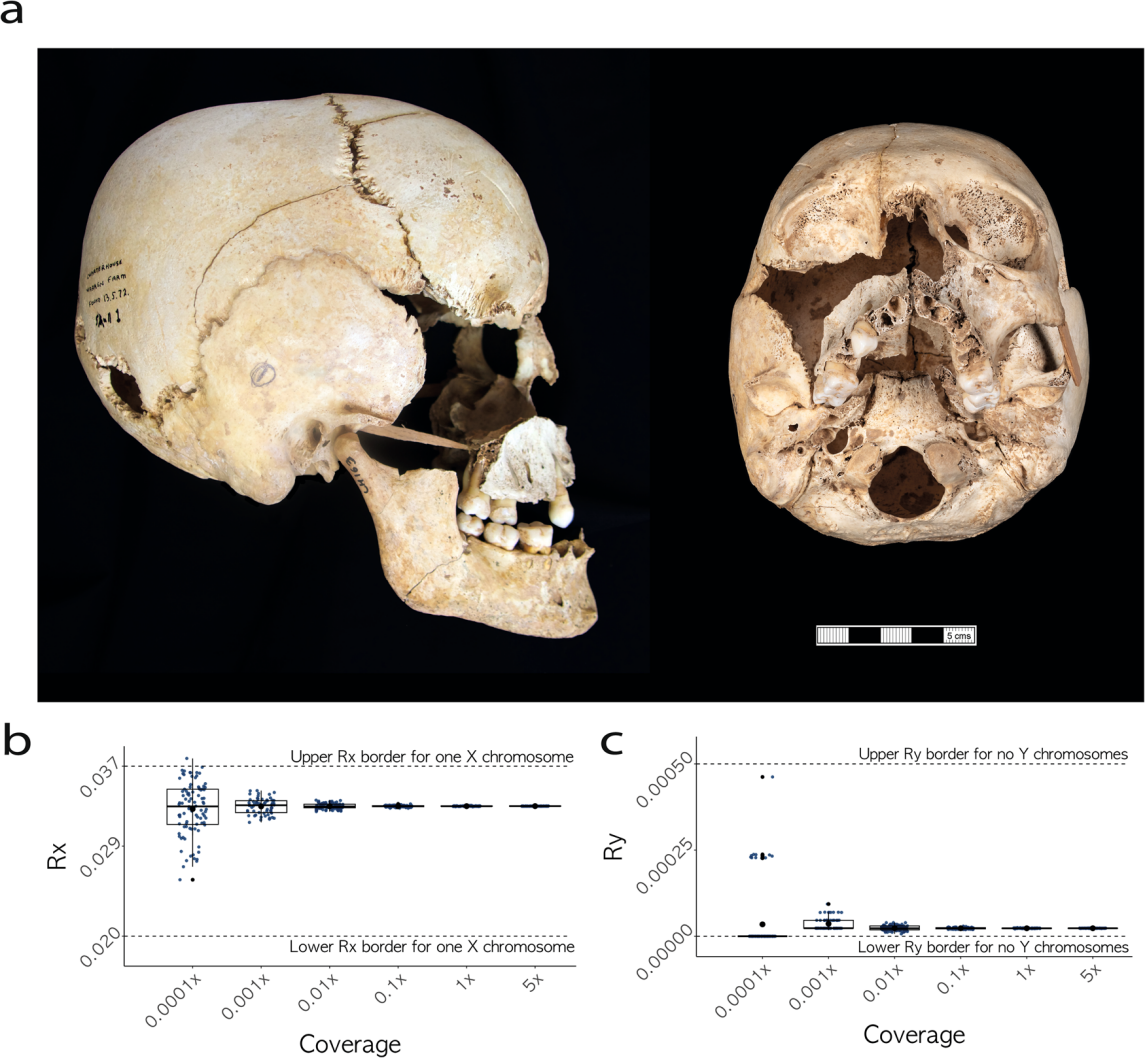
An individual with mosaic Turner syndrome from Iron Age Somerset, UK. **a** The cranium of the identified individual C10090 (CH163) from Charterhouse Warren (45,X0/46,XX) with mosaic Turner syndrome, exhibiting features consistent with a female morphological characterisation (full description in Supplementary Note 1). Scale bar indicates 5 cm. Image credits: Rick Schulting, Ian Cartwright. **b** *R*_x_ values for C10090 (45,X0/46,XX) across six different coverage levels (*n* = 507), dashed lines representing *R*_x_ boundaries for one copy of chromosome X. Mean is plotted as a circle, median as a horizontal line, lower and upper hinges correspond to the first and third quartiles, error bars represent ± 1 SD (Supplementary Data 4). **c** *R*_y_ values for C10090 (45,X0/46,XX) across six different coverage levels (*n* = 507), dashed lines representing *R*_y_ boundaries for no copies of chromosome Y. Mean is plotted as a circle, median as a horizontal line, lower and upper hinges correspond to the first and third quartiles, error bars represent ± 1 SD (Supplementary Data 4).

In order to confirm that the detected monosomy could not have been an artefact of poor DNA preservation, the high-coverage genome was downsampled to six different coverage levels 100 times to test the consistency and thresholds of the karyotypic assignment. As shown in Fig. 3b and c, the assignment remained consistent with a 45,X0 karyotype across coverage levels, even with as low as 0.0001x genomic coverage. The *R*_*x*_ and *R*_*y*_ values exhibited higher variability in lower coverage levels, as would be expected by the increasingly limited number of reads, but they remained within the established borders for one copy of X (Fig. 3b) and no copies of Y (Fig. 3c) chromosomes respectively.

### Detection of an Iron Age individual with Trisomy 21 (47,XY, + 21)

In addition to estimating the number of sex chromosomes in a karyotype, our approach can be applied to quantify the coverage of any chromosome by estimating the proportion of sequences aligning to that chromosome over the ‘autosomal baseline’ or *N*_a_ and thus also identifying autosomal aneuploidies. Indeed, a neonate with trisomy 21 was identified with this method (C10462, WS267 from Wetwang Slack) from Iron Age Yorkshire with a 47,XY, + 21 karyotype (Fig. 2, Supplementary Note 3 and Supplementary Fig. 2).

## Discussion

The identification of individuals with autosomal and sex chromosomal aneuploidies in the ancient genomic record contributes to a more detailed reconstruction of past societies and highlights the limitations of approaches focusing on binary classification of karyotypic sex. Examining the genomic information from these individuals in conjunction with osteological and archaeological data is crucial in providing a comprehensive perspective of aneuploidy across time.

Certain aneuploidies affecting the sex chromosomes can lead to disorders of sex development (DSDs) which result in mixed or ambiguous sex-related physical characteristics^36^. Many people with mild disorders experience few effects and are often unaware that they have a DSD. However, severe cases can produce stronger physical effects, including factors, which may be relevant to their own and society’s perception of their sex or gender^20^. There is evidence that people with DSDs exhibit elevated rates of gender dysphoria^37^, highlighting the potentially complex relationship between the physical manifestations of DSDs and a person’s sex and social gender as understood by themselves and their wider community^38–40^. It is difficult to know an ancient individual’s conception of their own gender identity and gender norms in the past may not align with those of the present day. However, while most people with sex chromosomal aneuploidies in the past may have lived their lives within conventional gender norms, it is possible that an elevated proportion would have been seen to transgress gender boundaries. Therefore, consideration of past people with sex chromosomal aneuploidies alongside accompanying gendered grave goods may provide a way of exploring past gender variability and how this may or may not relate to the physical effects of DSDs^20^. This idea was explored by Moilanen and colleagues^20^ in relation to an individual with possible Klinefelter Syndrome from early medieval Finland who was buried with both typically masculine- and feminine-gendered grave goods.

The three skeletons with Klinefelter syndrome from this study were buried in different contexts and time periods, but with certain similarities, the most important one being that their burials did not reveal any differences in how they were perceived by their contemporaries. The individual with Klinefelter syndrome from Wetwang Slack^41^ (C10427, Skeleton ID: WS224) was identified osteologically as male, around 18–19 years at death without associated grave goods. The individual found under Longwall Quad, Magdalen College (C11119, Skeleton ID: Sk4205) possessed sexually dimorphic features, which were consistently male; nearly all of them strongly male and two, probably male^42^. With an estimated stature of 176 cm (+/−3.27 cm), the individual was tall compared with the rest of the burial assemblage. The individual from Trinity Burial Ground (C11569, Skeleton ID: Sk30828) was an adolescent, found without additional artefacts and occupying a plain wooden coffin lacking metal fittings, in a part of the burial ground where many of the burials may have represented poorer members of society. By employing observations of epiphyseal fusion^43^ and dental development and eruption^44^, C11569 was estimated to have been between 16 and 19 years of age. Generally, many of the epiphyses were unfused and this suggested delayed skeletal development, or a prolonged growth period. Had the individual lived to attain skeletal maturity, this may have resulted in a taller-than-average stature. Similarly, the individual from Lincoln Eastern Bypass (C13582, Skeleton ID: 16586) with a 47,XYY karyotype had a stature of 184.2 cm, taller than average but within the male height range for the cemetery and was osteologically characterised as male^42^ (more details on the archaeological contexts and osteological assessment for all individuals in Supplementary Note 1).

Regarding the individual with mosaic Turner syndrome from Charterhouse Warren (C10090, Skeleton ID: CH163), it was possible to confidently match the cranium and mandible (the latter yielding the tooth analysed in this study), but no postcranial elements could be associated. Relatively few formal burials are known for the Early Iron Age of southern Britain, so it is difficult to assess how unusual Charterhouse Warren was for its period. Indeed, considerable variability seems to be the norm^45^ and disarticulated Iron Age human remains have been found in other caves in southwest England^46,47^. The main skeletal manifestation of Turner syndrome is short stature^48^, which could not be assessed given this lack of association. The clinical literature also records some changes in skull morphology^49^; while these are difficult to address given the skull’s incomplete and reconstructed state (see Supplementary Note 1), the cranium does appear to have a high-arched palate which is one of the skeletal changes seen in Turner syndrome (Fig. 3a). Morphological characteristics of the cranium including slight brow ridges, sharp orbital margins, rounded orbits, high frontal, and smooth nuchal area support a female biological sex estimation^42^ (Fig. 3a). The Charterhouse Warren individual died as a young adult, ca. 18–22 years old (see Supplementary Note 1). Interestingly, there is a reported relationship between spheno-occipital synchondrosis closure and the onset of puberty^50^, and in the Charterhouse Warren individual the spheno-occipital synchondrosis is unfused, implying that they would not have experienced menarche, despite their estimated age. A delay in, or absence of, menarche is a feature of Turner syndrome, caused by the absence or low levels of oestrogen^16,17^.

Identification of aneuploidies such as Down syndrome, which can result in neurodevelopmental problems^51^ can provide insights into care within ancient societies, as well as how people with these conditions, which have characteristic physical manifestations, were perceived by their peers. The individual with Down syndrome from Wetwang Slack^41^ (C10462, Skeleton ID: WS267) was a male neonate, buried in the ditch of a square barrow containing a primary adult female burial. However, the burial of neonates within the ditches of pre-existing barrows appears to have been relatively common at Wetwang Slack and is not likely to relate directly to the genetic condition of this individual.

Overall, individuals with aneuploidy from this study were largely buried in accordance with the customs that prevailed in their lifetimes, with the exception of CH163 from Charterhouse Warren (C10090), suggesting that they were most likely considered ordinary members of their community. Inferences regarding their view of themselves and their gender are limited, not least due to the lack of associated gendered grave goods or records. Of course, our understanding of attitudes on identity and gender in Medieval England, surpasses that of the Iron Age, primarily due to the abundance of historical records available for the former. Thus, while patriarchal stereotypes and strictly defined gender roles prevailed in the Medieval period^52^, female individuals from the Iron Age have intriguingly often been found in elite burials with “objects of power” typically associated with male occupants^53,54^. An additional aspect to consider, beyond gender identity, is the fact that people with sex chromosomal aneuploidies are substantially more likely to experience later onset of puberty and childlessness^55^ and the influence these would exert on their daily lives. Indeed, there were osteological indications of delayed growth in one of the three individuals with Klinefelter (C11569), as well as in the individual with mosaic Turner syndrome (C10090) and none of the six individuals in this study were buried in proximity to progeny, although this is not a rare occurrence. Lastly, we would advise caution when interpreting the frequency of aneuploidies in the past, as certain aspects of ancient DNA studies could introduce bias and influence our observations; namely the preferential targeting of crania (often treated differently to postcranial elements in mortuary contexts) due to better DNA preservation^56^, the scarcity of skeletal material covering certain periods and regions (for example due to widespread cremation) or variable infant representation in skeletal assemblages^57^.

The method presented here provides a straightforward approach that can identify chromosomal sex as well as autosomal and sex chromosomal aneuploidies (XXY, X0, XYY, XXX and +21) on genomic data of varying quality, while also flagging up likely contamination. In principle, our method can be expected to detect around half of contamination scenarios, i.e. those cases when the contamination originates from an individual with a different chromosomal sex to the individual under study. One individual from a published study (VK125^21^, who was excluded from downstream analysis in the original paper due to low coverage) had intermediate values both for *R*_x_ and *R*_y_ (Fig. 1a), indicating contamination with modern-day DNA from someone with different chromosomal sex to the individual under study, which could be relatively common in low-yield ancient DNA samples that have been handled extensively^58–60^. The presence of different-sex contamination tends to inflate the observed *R*_x_ values while decreasing *R*_y_ values for XY individuals and inflate the *R*_y_ values while decreasing *R*_x_ values for XX individuals, due to the presence of additional sets of autosomes in the denominator. Importantly, these values are different from those observed in any type of sex chromosomal aneuploidy and thus they can be distinguished with this method, unlike previously^8^. We provide the classification of putatively contaminated libraries in our implementation of the methods in this paper. A potential limitation of the method is its lack of sensitivity to structural variation, such as micro-deletions in the Y chromosome^61^. Rare cases of aneuploidy that can be attributed to chromosomal rearrangements^18^ can also not be excluded, as they cannot be identified by this method currently.

As indicated from our results, the ever-increasing number of ancient genomes will only multiply the opportunities to observe and contextualise genomic diversity and more importantly, methods like the one presented here will provide another layer of information that can contribute to a more detailed reconstruction of the human past.

## Methods

### Ancient DNA extraction and sequencing

For the genomic data generated in this study, DNA was extracted from auditory ossicles, dentine or the petrosal part of the temporal bone^62^ and double-indexed single-stranded libraries were prepared and amplified using Agilent Bravo Workstations^63,64^ before sequencing approximately 2.5–5 million read-pairs per library on the Illumina NextSeq 500 platform (with the exception of C13582 that was sequenced directly on the Illumina NovaSeq 6000 to approximately 70 million reads). The same libraries, except C13582, were then sequenced for a second round on the Illumina NovaSeq 6000 to increase the sequencing depth per library, obtaining a final average coverage of ~0.85x, except in the case of C10090 which was sequenced to 9.8x coverage.

### Bioinformatic processing

Sequenced libraries were preprocessed and analysed using the nf-core/eager pipeline^65^ version 2.3.3 and the version-matched Singularity image. The hs37d5 genome and version 50.0 of AADR (v50.0_1240K)^66^ were used as references. Read trimming and adapter removal was applied within the pipeline with the following options specified:

“--complexity_filter_poly_g”,

“--clip_forward_adaptor AGATCGGAAGAGCACACGTCTGAACTCCAGTCAC”,

“--clip_reverse_adaptor GGAAGAGCGTCGTGTAGGGAAAGAGTGT”,

“--preserve5p” and “--clip_readlength 35”.

Paired reads were collapsed (“--mergedonly”) and aligned using bwa aln^67^ with maximum edit distance 0.01 (“--bwaalnn 0.01”) and subsequently filtered (“--run_bam_filtering”). PCR duplicates were identified and removed with DeDup^68^ (“--dedupper dedup”). Contamination estimates based on heterozygosity on the X chromosome were calculated using ANGSD^29^ and contamination estimates based on mitochondrial DNA were calculated using schmutzi version 1.5.6^69^.

Y-chromosome haplogroups were inferred with Yleaf version 3.1^70^, using only reads with mapping quality over 30. To determine mitochondrial haplogroups, the deduplicated BAM files output by nf-core/eager were first filtered for mitochondrial reads and realigned using bwa aln (“-n 0.01”, “-l 1024”, and ”-k 2”) to rCRS (rCRS_NC_012920). Variants in mitochondrial reads were identified with BCFtools version 1.10.2^71,72^: BAM files were filtered with “bcftools view” for minimum depth 1 (“--include ‘FMT/DP > = 1”) and variants called with “bcftools call” specifying “--multiallelic-caller”. Mitochondrial haplogroups were then assessed using “haplogrep classify” version 2.2.8^73^ with the best 3 hits calculated (“--hits 3”).

### Published data

Published data^21,28^ for 570 individuals were downloaded from the European Nucleotide Archive.

### Sex identification

Sex identification was done using the method reported in this study. The karyotype assignment thresholds were defined separately for shotgun sequenced and for target-enriched libraries and details on their determination can be found in Supplementary Note 4. BAM files for both published and newly generated genomes were filtered for mapping quality (-q30) using SAMtools version 1.13^74^ and the sex identification script (karyo_RxRy_script.py, from https://github.com/kyriaki-anast/karyo_RxRy) was applied using this command:

“samtools view -q30 bam_file.bam | python2 karyo_RxRy_script.py > output_file”.

### Chromosomal coverage

Chromosomal coverage for all newly published individuals (Fig. 2) was calculated by counting the number of sequences mapping to the human reference genome hs37d5, filtering only for regions included in the 1000 Genomes Strict Mask^75^. The counts of sequences mapping to each chromosome were then normalised between chromosomes and across individuals to account for differences in sequencing effort per library.

### Downsampling

The high coverage genome (9.8x) of individual C10090 was randomly downsampled to six different coverage levels, in 100 independent repetitions for each level. Final genomic coverage levels were expressed as proportions of initial coverage. The sex identification method was applied to all downsampled BAM files as described above (applying a mapping quality filter (-q) of 30). Raw output is in Supplementary Data 4 and data is plotted in Fig. 3b and c.

### Statistics and reproducibility

Sample sizes were not determined in advance. Methods were applied to published data generated from different groups to control for batch effects. Raw data, analysis output, newly developed scripts and software versions have been made available.

### Ethics declaration – sample provenance

Permissions to study the archaeological samples presented in this study were obtained as follows. CH163 - Permissions acquired from David Walker, archaeological curator at Wells Museum. WS 224 (Gen Lab 199) and WS 267 (Gen Lab 162) - Permissions acquired from Jo Buckberry, Reader in Biological Anthropology at the University of Bradford. Sk 4205 - Permissions acquired from Louise Loe, head human osteologist at Oxford Archaeology South and David Radford, archaeologist at Oxford City Council. Sk 30828 - Permissions acquired from Louise Loe, head human osteologist at Oxford Archaeology South and Stephen Rowland, project manager at Oxford Archaeology. Sk 16586 – Permissions acquired from Malin Holst, Managing Director at York Osteoarchaeology. Minimally destructive sampling for aDNA analysis followed guidelines issued by the Department for Culture, Media and Sport (DCMS) and the Advisory Panel on the Archaeology of Burials in England (APABE) (apabe.archaeologyuk.org).

### Reporting summary

Further information on research design is available in the Nature Portfolio Reporting Summary linked to this article.

### Supplementary information

Supplementary Information

Description of additional supplementary files

Supplementary Data 1-4

Reporting summary

## Data Availability

Sequencing data (BAM files) are available on the European Nucleotide Archive (accession code: PRJEB65239). Public datasets^21,28^ were accessed through the European Nucleotide Archive (accession codes: PRJEB37976 and PRJEB32566). Source data used to plot Fig. 1a are in Supplementary Data 2. Source data used to plot Fig. 2b, c are in Supplementary Data 4. Source data used to plot Fig. 3 are in Supplementary Data 3.

## Code availability

The sex identification method^76^ described and used in this paper can be accessed at https://github.com/kyriaki-anast/karyo_RxRy. This includes an option for target-enriched libraries and autosomal aneuploidy detection.
