# Supplementary material for: Detection of chromosomal aneuploidy in ancient genomes
Source: Commun Biol. 2024 Jan 11;7:14. doi: 10.1038/s42003-023-05642-z (PMC10784527; doi:10.1038/s42003-023-05642-z)
Supplement: Supplementary file 1 — Supplementary Information [file 42003_2023_5642_MOESM1_ESM.pdf]

Supplementary Information

Detection of chromosomal aneuploidy in ancient genomes

Kyriaki Anastasiadou[1]*, Marina Silva[1], Thomas Booth[1], Leo Speidel[1,2], Tony Audsley[3], Christopher Barrington[4], Jo Buckberry[5], Diana Fernandes[6], Ben Ford[7], Mark Gibson[7], Alexandre Gilardet[1], Isabelle Glocke[1], Katie Keefe[8,9], Monica Kelly[1], Mackenzie Masters[8,10], Jesse McCabe[1], Lauren McIntyre[7], Paola Ponce[8,10], Stephen Rowland[7], Jordi Ruiz Ventura[8], Pooja Swali[1], Frankie Tait[1], David Walker[11], Helen Webb[7], Mia Williams[1], Annsofie Witkin[7], Malin Holst[8,10], Louise Loe[7], Ian Armit[10], Rick Schulting[12], Pontus Skoglund[1]*

*Corresponding authors: kyriaki.anastasiadou@crick.ac.uk, pontus.skoglund@crick.ac.uk

1. Ancient genomics laboratory, The Francis Crick Institute, London, United Kingdom
2. Genetics Institute, University College London, London, United Kingdom
3. Independent Scholar, Wells, United Kingdom
4. Bioinformatics and Biostatistics Science Technology Platform, The Francis Crick Institute, London, United Kingdom
5. School of Archaeological and Forensic Sciences, University of Bradford, Bradford, United Kingdom
6. Network Archaeology, Lincoln, United Kingdom
7. Oxford Archaeology, Oxford, United Kingdom
8. York Osteoarchaeology, York, United Kingdom
9. On-Site Archaeology, York, United Kingdom
10. Department of Archaeology, University of York, York, United Kingdom
11. Wells and Mendip Museum, Wells, United Kingdom
12. School of Archaeology, University of Oxford, Oxford, United Kingdom

Supplementary Note 1: Archaeological contexts and osteological assessment

1.1.Charterhouse Warren

Authors: Rick J. Schulting and Louise Loe

Skeleton CH163- Lab Code C10090 - Karyotype (45,X0/46,XX)

CH163 is the partial skull of a young adult female. It had been reconstructed by the excavators prior to our examination. The calvarium is largely complete, with the exception of a missing section of the anterior left parietal. The maxillofacial region is less complete, missing the right nasal bones, both ethmoid and zygomatic bones, and the sphenoid. The right hemi-mandible is complete apart from a damaged coronoid process. The left hemi-mandible is missing part of the interior body and much of the ascending ramus. A large number of Wormian bones are present on the occipital.

There is a well-documented association with chronic intestinal haemorrhage[1], in turn linked with anaemia[2] and the orbits of the C10090 cranium show marked pitting known as *cribra orbitalia* (Figure 3a), widely interpreted as a response to anaemia. This is usually attributed to insufficient dietary iron, but other causes can include thalassemia and high parasite loads[3]. A possible link between *cribra orbitalia* and Turner syndrome has also been noted in a suspected case from early-twentieth-century Portugal, though the diagnosis has not been confirmed genetically[4].

The maxillary right canine and all molars are present apart from the right first molar. The three mandibular right molars and the left second molar are present. All other teeth have been lost post-mortem. First and second molars show moderate cusp wear with no dentine exposure. The third molars are minimally worn.

The eruption and state of wear of the teeth suggests that the skull is that of a young adult. The third molar roots are fully formed and the apex appears to be closed, though slight damage at the visible root tip makes this uncertain. This together with its minimal wear would suggest an age ca. 18-22 years[5,6]. However, the spheno-occipital synchondrosis (SOS) is unfused, which would normally indicate an age of no more than 14–16 years in females[7–10] (though see[11] who note that some studies support later fusion). This age discrepancy may be another effect of Turner syndrome, in which skeletal growth (including fusion) may be delayed relative to dental development[12,13]. Furthermore, the relationship between SOS closure and the onset of puberty[7] implies that the Charterhouse Warren individual would not have experienced menarche, despite her age. A delay in, or absence of, menarche is a feature of Turner syndrome, caused by the absence or low levels of the growth hormone oestrogen[14,15].

There is an irregular, narrow oblong perforation to the mid-frontal bone that is interpreted as post- rather than peri-mortem in origin. While there is some internal bevelling, the radiating fractures have transverse fracture margins that statistically are more often seen in dry bone[16,17].

There is mild healed porotic hyperostosis on the posterior frontal and anterior parietal bones (stage 1 in [18]). The roofs of both orbits show marked porosity typical of *cribra orbitalia*, extending onto the orbital notches (stage 4 in [18]).

Radiocarbon dating

Author: Rick J. Schulting

A sample (0.68g) of the mandible containing the tooth used in the genomic analysis (C10090) was taken for AMS (accelerator mass spectrometry) radiocarbon dating. Pre-treatment followed the standard protocols in place at the Oxford Radiocarbon Accelerator Unit in the School of Archaeology, University of Oxford[19]. The results meet the quality control criteria for well-preserved collagen[20,21] (Supplementary Table 1). The radiocarbon determination of 2456 ± 26 BP yields a calibrated date range of 2703–2365 cal BP or cal BCE 754–416 (95.4% confidence), placing it in the Early Iron Age. The stable carbon isotope (d^{13}C) value of −20.9‰ is typical of a terrestrial diet.

Supplementary Table 1. Radiocarbon dating and associated quality control data for bone collagen from Charterhouse Warren mandible (CH163). Calibrated in OxCal v.4.4[22] using Intcal20[23].

Lab code	Date ^{14}C yr	cal BP (95.4%)	d^{13}C$_{VPDB}$	C:N	% collagen yield
OxA-37856	2456± 26	2703–2365	-20.9‰	3.3	1.2

1.2.Wetwang Slack

Author: Jo Buckberry

Wetwang Slack is the largest Iron Age cemetery excavated in England to date. Located in East Yorkshire the extensive square barrow cemetery contains c.450 burials (numbered consecutively) and is located in a larger funerary landscape. Two individuals from this cemetery population are discussed in this manuscript

Skeleton Wetwang Slack 224 – Lab Code C10427 – Karyotype (47,XXY)

Wetwang Slack 224 was almost complete, with some small bones missing. The skull and ribs were fragmentary, and several long bones were broken. As the long bones were not fully fused, age was estimated using epiphyseal and apophyseal fusion and dental development[5,24,25]. The greater trochanter of the femora and distal tibiae were in the process of fusing, suggesting an age between 14 and 16 years. Complete fusion of the lesser trochanter of the femur suggests an age of at least 16 years. The distal radii, distal ulnae, distal femora, distal tibiae and distal fibulae were all unfused, suggesting an age below 18-20 years. Combined, the fusion data suggests an age of 16-18 years. Dental radiographs showed the second molars were all complete, whereas the third molars had complete roots, but no evidence of closure of the apex. This provides an age estimate of either 17.5 years[26] or 18-19 years[5]. As dental development is less likely to be affected by external factors[28] and the AlQahtani et al method[5] has been shown to perform well in tests of different methods[30], the slightly older age estimate of 18-19 years is most appropriate for this individual.

As the long bones were not fully fused, sex was assessed using the features of the pubic bone[31,32], as these features and especially the ventral arc have shown to be reliable for adolescents[33]. Other features of the pelvis[29] were considered to support the assessment but not replied upon, due to the age of the individual. Likewise, features of the skull[24,34] were not considered, as these have not been established for older adolescents, and the crania of all young adults tend to be more gracile. All three Phenice (1969)[35] traits suggested this individual was male. This was supported by a narrow sub-pubic angle and moderately narrow greater sciatic notch. Thus, this individual was sexed (skeletally) as male.

Skeleton Wetwang Slack 267 – Lab Code C10462 – Karyotype (47,XY,+21)

Wetwang Slack 267 comprises the incomplete and fragmentary remains of an infant, represented by a large number of cranial fragments and two long bone shaft fragments more likely to be from the femora, tibiae or humeri than the radii, ulnae or fibulae. The sizes suggest two different bones were present, possibly a femur and a humerus. Three dental crowns were present. It was not possible to ascertain if these were mandibular or maxillary teeth, due to the early stages of development. The canine and first molar were assessed as being between cusp outline complete and crown half complete; the second collar was assessed as being between coalescence of cusps and cusp outline complete. This provides and age estimate of below 0.2 years[36] and birth plus or minus 1 week[5]. As the latter method was developed using more fetal and neonatal individuals and included both maxillary and mandibular teeth, the younger age estimate is preferred. This suggests Wetwang Slack 267 was a neonate / full term infant. No attempt was made to sex this individual as methods of sex assessment for non-adults are generally regarded as less reliable than those for adults[38].

1.3.The Medieval Cemetery under Longwall Quad, Magdalen College, Oxford

Authors: Helen Webb, Louise Loe

Skeleton 4205 - Lab Code C11119 - Karyotype (47,XXY)

Skeleton 4205, was one of 115 discrete, articulated skeletons, excavated from a 12th-13th century Christian cemetery by Oxford Archaeology during redevelopment of the library at Magdalen College, Oxford[31,39,40]. The skeletons were found under Longwall Quad and have been interpreted as coming from the cemetery attached to the Hospital of St John.

Like the other skeletons in the cemetery, and in keeping with Christian burial tradition, 4205 was recovered from a plain earth cut east-west orientated grave. The grave was located towards the southern limit of the cemetery and the skeleton was lying in a supine (on back) position with their arms and legs extended and their head positioned on its left side facing north. It is not possible to draw any conclusions regarding the significance of the position of the grave because only the south-west corner of the cemetery was revealed during the excavation. A small number of residual pot and animal bone fragments was recovered from the backfill of the grave, but there were no artefacts associated with the burial, nor was there any evidence for a coffin. Although no shroud pins or other evidence

was found, the individual had probably been buried in a shroud or winding sheet, as was common for the period[29,31].

Skeleton 4205 was approximately 90 percent complete with only bones from the feet missing. The bones were relatively intact, with surfaces which exhibited slight, patchy erosion that penetrated the bone surfaces in some areas (consistent with Grade 2 after [35]). Overall, the skeleton was in good condition.

Based on sexually dimorphic traits of the skull[41] and pelvis[41] the skeleton was estimated to be male. Eight out of ten of the cranial traits were of definite male expression, while two (frontal slope and mandibular ramus width) were consistent with male features but were not as clearly expressed (these were recorded as 'probable male'). Similarly, of the six observable pelvic traits (seven pelvic features were not recordable), five were definitely male and one, the ilium auricular surface, was probably male. Sexually dimorphic traits of mixed expression, as described here, are typically encountered in most skeletons from all time periods[42].

The dimensions of the femoral (50.0mm) and humeral heads (47.5mm) were employed as secondary indicators of osteological sex (with reference to [43,44]). Both measurements are within the male ranges[45,46] (humeral head diameter >47mm, femoral head diameter >48mm). Based on morphological changes on the pubic symphyses[47] and auricular surfaces[48,49] of the pelvis, as well as the level of molar attrition[50], the skeleton was aged as a middle (36-45 years) or mature adult (45+ years), possibly as young as 36 years but likely over 45 years at death.

The maximum length of the left femur (48.2cm) applied to Trotter's regression formula for white males[51] indicated that the stature of Skeleton 4205 was 176.13 cm (+/- 3.27cm), or 5 ft 9.34 in. This is taller than the average male stature of 171.50cm (based on 16 males), calculated for the assemblage and the third tallest male stature in the assemblage. Although not exceptionally tall (the skeleton does not classify as of clinically tall stature because it is not taller than 2SD above the mean for the male sample – this would be 182.4cm (SD is 5.45)), the taller than average stature may be significant in terms of the diagnosis of Klinefelter syndrome, considering one of the classic, physiological signs of the condition is taller than average height[52].

Pathology included minor, well healed inflammatory lesions (periostitis) on the tibiae (shin bones), a well healed fracture of the fourth left metacarpal (hand bone) and lesions pertaining to joint disease in the spine (Schmorl's nodes, marginal osteophytes, spondylosis deformans and osteoarthritis) and hip joints (osteoarthritis), which are particularly common in mature adult skeletons, such as this. In addition, calculus (mineralised plaque) deposits, carious lesions and dental enamel hypoplasia (DEH) were present, each of these involving multiple teeth. The DEH, which appears as lines, pits or grooves in the dental enamel, represents an interruption of the enamel development during childhood[53] and results from prolonged periods of illness and/or malnutrition[54]. The teeth affected, and the positions of the DEH lesions, indicated that the individual had suffered, and recovered from, repeated episodes of physiological stress between the ages of three and 11 years[54].

Radiocarbon dating

A sample of the right femur shaft was taken for AMS (accelerator mass spectrometry) radiocarbon dating. Pre-treatment followed the standard protocols in place at the AMS Facility Scottish Universities Environment Research Centre (SUERC)[55]. The results are quoted in line with the international standard known as the Trondheim Convention[56]. The results meet the quality control criteria for well-preserved collagen[55] (Supplementary Table 2). The radiocarbon determination of 857 ±28 BP yields a calibrated date range of cal AD 1200–1280 (68.2%) or cal AD 1050–1080 (0.7%); 1150-1290 (94.7%). The stable isotope data from the human bone collagen suggests that the individual may have had a marine component in their diet, and based on the stable carbon isotope ($\delta^{13}C$) value of −19.7‰ and the method of Arneborg et al.[56] using end-members of -21.0‰ (fully terrestrial) and -12.5‰ (fully marine), a 15% marine contribution (±10%) has been determined. This equates to a regional marine offset (ΔR) of 0 ± 50 years which has been used in the calibration, a mix of the IntCal13 and Marine13 calibration curves[57].

Supplementary Table 2. Radiocarbon dating and associated quality control data for bone collagen from Longwall Quad, Magdalen College femur shaft (Skeleton 4205). Calibrated in OxCal v.4.3.2[38] using Intcal13, LocalMarine,15, 10)[25].

Lab code	Date ^{14}C yr	±	cal AD (95.4%)	$\delta^{13}C_{VPDB}$	C:N	% collagen yield
SUERC-80255 (GU47916)	857	28	1050-1290	-19.7‰	3.2	4.4

1.4. Trinity Burial Ground, Hull

Authors: Annsofie Witkin, Louise Loe and Stephen Rowland

Skeleton 30828 - Lab Code C11569 - Karyotype (47,XXY)

Trinity Burial Ground (1785-1861) was an overflow cemetery, established due to concerns that the original burial ground, attached to Holy Trinity Church (now Hull Minster), would reach full capacity[25]. At least 8,791 individuals and an extensive assemblage of artefacts and coffin fittings across an area of almost 3,000m^2, representing approximately 40% of the burial ground, were excavated by Oxford Archaeology in 2020-2021 to allow for major infrastructure improvements.

Skeleton 30828 was estimated to be nearly complete with only a few epiphyses and bones from the hands and feet missing. Some areas had undergone post-mortem breakage, but this was largely limited to the ends of the diaphyseal shafts and the cranium. Most of the cortical surfaces were affected by slight erosion (scored as a grade 3, after McKinley 2004, 16[38]). Overall, the skeleton was judged to be in a fair condition.

The age of the individual was assessed by observing epiphyseal fusion[25] and dental development and eruption[5]. The epiphyses suggested that the individual was between 11 and 20 years of age, probably the lower end of this range, considering most epiphyses were unfused. The stage of dental development and eruption suggested an age of between 16 and 19 years. Considering that dental development and eruption correlate more closely with chronological age than epiphyseal fusion does[25], the likely age of the individual is 16-19 years. The general lack of epiphyseal fusion suggests delayed skeletal development, or a prolonged growth period which may have resulted in a taller than average stature, had the individual lived to attain skeletal maturity.

Relatively minor pathological conditions were present. These comprised two midline developmental abnormalities, including retention of the midline frontal suture (a metopic suture, often recorded as a non-metric trait[58]) and a first cervical vertebra with a cleft neural arch[59]. Both abnormalities are relatively common in archaeological populations and would not have had any adverse effect on the individual in life (who probably wasn't aware of them).

In addition to developmental abnormalities, bilateral type 2 *cribra orbitalia* (scattered fine foramina[3]) and bilateral *cribra femoralis*[60], were also present. These conditions may refer to iron deficiency anaemia[61], but this is not certain. The aetiology of cribrous lesions such as these has been the subject of considerable debate in recent years, and there are several possible explanations, vascular imprints being one of them[62]. In the present case, the fact that the lesions are present on both orbital roofs and the femora, could suggest that iron deficiency anaemia is a more likely cause.

Another pathological condition observed in the skeleton was unilateral maxillary sinusitis (bone inflammation in the sinuses). The lesions were healed and not active at the time of death. Maxillary sinusitis may be caused by dental disease (for example, a dental abscess) or by irritants (e.g. smoke/pollution) in the environment[63], the latter being the most likely explanation for the lesions in the present skeleton.

Lastly, all of the first molars and the right mandibular second molar had medium to large carious lesions.

1.5.Lincoln Eastern Bypass, Lincoln

Authors: Katie Keefe, Paola Ponce, Jordi Ruiz Ventura and Malin Holst

Skeleton 16586 (135) - Lab Code C13582 - Karyotype (47,XYY)

A total of 734 skeletons were excavated by Network Archaeology from 2016 to 2019 prior to the construction of the Lincoln Eastern Bypass, likely dating from AD 680 to 890. The skeletons were buried in west-to-east orientations in supine extended positions, following Christian tradition, in earth-cut graves, with evidence of oak coffins in some graves.

Skeleton 16586 was interred at the western edge of the southwestern part of the cemetery in the standard position and alignment.

Skeleton 16586 was 90% complete (missing the left hand, both feet and most ribs), moderately fragmented, with moderate surface preservation (Grade 3, McKinley, 2004, 16[38]). All sexing criteria of the skull, pelvis and supplementary skeletal measurements suggested the individual was male[29,31,64]. Based on the ageing criteria of the pubic symphysis and auricular surface[42,43,64] the skeleton was a mature adult, aged 46 years old or older. The individual was 184.2cm tall[47], which was much taller than the male mean from the site at 171.1cm, and also than the male average (172cm) for the period[65], but was within the male stature range for the cemetery.

The skeleton had a number of minor non-metric traits that were assessed following[31,58,66]. These included an ossicle in lambdoid, an accessory lesser palatine foramen, bridging of the supraorbital notch, plaque, exostosis in the trochanteric fossa and a third trochanter.

The individual had bilateral shallow, almost imperceptible fovea capitis of the femoral heads, where the ligament for the head of the femur attached, as well as an incomplete vertebral border shift at the sacro-lumbar border, indicating a developmental defect that

had occurred during the first month of gestation[59]. The first sacral vertebral body was fused to the remainder of the sacrum, as were the ala, however, the vertebra retained its inferior articulating facets, which had not been integrated into the remainder of the posterior sacral surface. The second sacral vertebra likely had superior articulating facets that corresponded with the facets on the first sacral vertebra, but unfortunately, this was not recovered.

Dental enamel hypoplasia lesions were recorded in ten of the 27 teeth. The presence of lines on the crowns of teeth as seen in the dentition of this individual occurs as a result of defective formation of tooth enamel during growth[50], following periods of physiological stress such as malnutrition or disease during the first seven years of childhood[67].

The individual had evidence for os acromiale in the left acromion of the scapula. The lateral end of the acromion was foreshortened and flattened, with a porotic surface. The presence of os acromiale has been linked to severe stress on the rotator cuff muscles of the shoulder during growth, which in turn prevents the natural fusion of the tip of the acromion with the acromial end. The presence of os acromiale in medieval populations has been suggested to be linked to intensive archery practice from a young age[51,68,69]. However, Mann and Hunt[70] have suggested there may be a genetic component to the condition.

A possible healed crush fracture was observed in the body of the first lumbar vertebra; the anterior body height measured 23.7mm and the posterior body height measured 30.8mm superior-inferiorly. The inferior and superior surfaces of the body were both convex. These fractures are usually caused by landing on the feet after a fall from a height, although in elderly individuals a fall onto the bottom can also lead to a crush fracture[71].

Schmorl's nodes were recorded in the thoracic and lumbar spine. Degenerative joint changes were observed in the thoracic, lumbar and sacral vertebrae, and in both manubrio-clavicular joints. Both conditions affect the joints of the spine and extraspinal areas of the skeleton due to physical activity, occupation, workload and advancing age[51,64].

The individual had widespread calculus concretions on 26 of 27 teeth and four caries lesions. One tooth that was lost ante-mortem, while the individual also had an impacted canine, two externally draining abscesses and moderate to severe periodontal disease. Dental calculus (dental plaque), dental infections (abscesses) and the exposure of roots of teeth due to receding gums are commonly observed in archaeological populations of all periods. These conditions have been linked to poor dental hygiene, diet and heavy wear of the teeth[50,51]. Impacted teeth can develop due to a genuine failure of the tooth to develop (congenital absence), or because the tooth develops but fails to erupt (impaction)[50] as occurred with this individual whose canine was visible in the crypt.

The skeleton was buried 10m to the southwest of a young middle adult of indeterminate sex, with a very tentative diagnosis of Klinefelter's Syndrome, based on a congenital radial head dislocation, though this may also be associated with other syndromes, including Apert syndrome, Ehlers-Danlos syndrome, Nail-Patella syndrome, Klippel-Feil syndrome and kyphosis.

Supplementary Note 2: Mosaicism investigation

a).

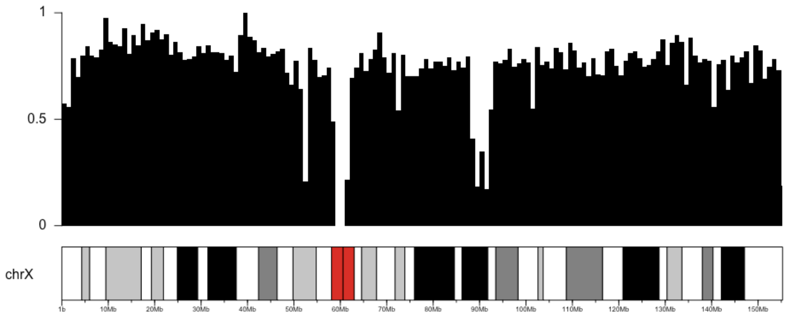

b).

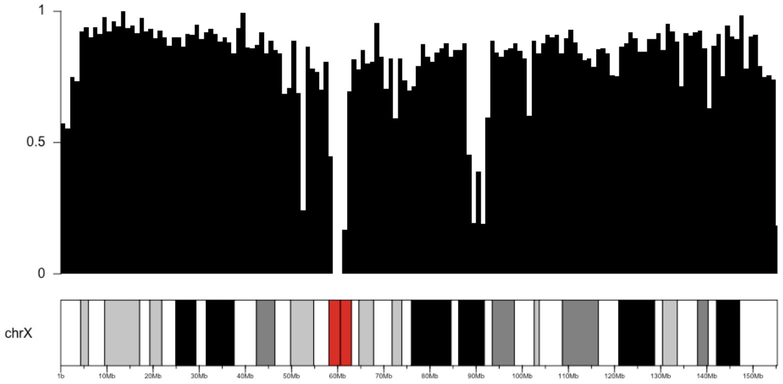

Supplementary figure 1: Sequencing read density across chromosome X. a). Read density plot of chromosome X for individual C10090 showing relatively even coverage across the chromosome (except for regions with mapping issues, like those harbouring repetitive elements), similar to chromosome X coverage for typical 46,XX individuals (b), using the R package (karyoploteR) https://github.com/bernatgel/karyoploteR .

Supplementary Note 3: Autosomal aneuploidies

The presence of additional autosomal chromosomes in the karyotype can be readily identified by counting the number of sequencing reads mapping to chromosome 21 and dividing this with the (previously defined) N_a to obtain an R_{21} estimate, equivalent to R_x and R_y for sex chromosomes. In this case, and given the higher frequency of chromosome 21 aneuploidy compared to other autosomal aneuploidies, we included the estimation of R_{21} as an optional but ready-to-use option of our script. Specifically, by adding option --chr21 as shown below, the output will include not only details about the biological sex and potential aneuploidies on the sex chromosomes but also the value for R_{21}, serving as an indicator of the presence of an additional copy of chromosome 21. We propose the use of 0.02 as a threshold and suggest investigating potential aneuploidies further (for example by restricting the analysis to authentic ancient DNA reads using PMDtools[72]. The proposed command is:

```
samtools view -q 30 bamfile.bam | python2 karyo_RxRy.py --chr21
```

The output for R_{21} of all 576 individuals used in this study can be seen in Supplementary Figure 2 below, where R_{21} for individual C10462 (Skeleton ID: WS 267) from Wetwang Slack was clearly above the proposed threshold.

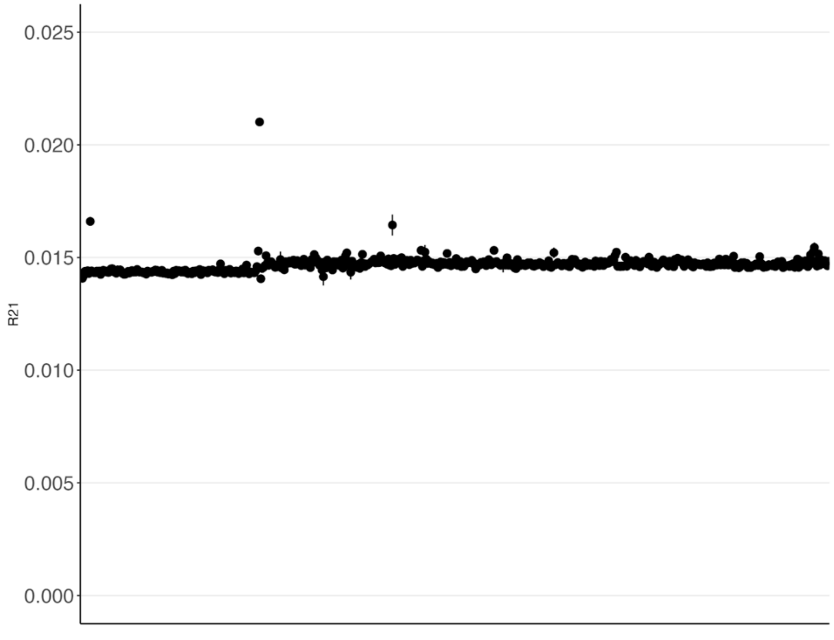

Supplementary figure 2: R_{21} values for 576 individuals included in this study. The R_{21} value above the proposed threshold of 0.02 belongs to individual C10462 (Skeleton ID: WS 267) from Wetwang Slack who was identified with three copies of chromosome 21, suggesting Down syndrome. Error bars represent Standard Error for R_{21}, as calculated by karyo_RxRy.py using binomial approximations.

Supplementary Note 4: Determining assignment thresholds

Our method focused on using the ratio representing the number of reads mapped on each sex chromosome over the number of reads mapped on the autosomes (excluding chromosomes 13, 18 and 21). These numbers are dependent on the fraction of the chromosome that can be best aligned to the reference and in order to get an estimate of that, we used the 1000 Genomes Project “Strict Callable Genome Mask”[73]. This Strict Mask filters for the genomic positions that represent the “accessible genome”, those unique regions of the genome where mapping is expected to be more accurate and pass certain quality filters (see original publication SI 9.2 “Callable genome mask” for detailed filters).

The sum of base pairs included in this Strict Mask on all autosomes (excluding 13, 18, and 21), i.e. the number equivalent to N_a in our method, was N_a = 1,899,515,379. Dividing the number of reads mapping to chromosome X with this number, we obtained a theoretical value of R_x = 0.0578 and dividing the number of reads mapping to chromosome Y with this number, we obtained a theoretical value of R_y = 0.004. With those values as starting points, we applied our method to calculate R_x and R_y for a large number of published shotgun genomes[74,75] and investigated their distribution compared to the expected theoretical values. For chromosome X, R_x values indeed clustered around those expected values for one or two chromosomes X, with a degree of variability that seems to be also present in modern genomic data[76]. Conversely, R_y values were 40% smaller in empirical data than the expected theoretical value of R_y = 0.004. However, since the complete absence or presence of a Y chromosome was clear even when R_y values were smaller than the theoretical value in the empirical data, the lower border was introduced based on the distribution of the empirical data (Figure 1a).

The choice of all autosomes (excluding 13, 18 and 21) as a baseline was made to account for variation in the autosomes, since if we only used sex chromosomes or one autosome to define our statistic, we would expect the results to exhibit even more variation. The dispersion in our plot can be explained to an extent by the variation in human genome sizes, as there seems to be up to 6% size difference in human genome sequences[77]. Repetitive elements (particularly LINE-1) seem to be responsible for most of this variation but it is noteworthy that chromosome Y is particularly rich in repetitive elements, like Copy Number Variants (CNVs)[78–80], while exhibiting substantial variation both in size and structure between individuals[81]. Therefore, methods aiming to quantify the number of sequences mapping per chromosome, a statistic that is reliant on genome size and the presence and number of repetitive elements, do seem to exhibit variation in their dispersal (see also for non-ancient DNA data[76,82].

Target enrichment (Capture) on 1240k positions

We have developed an adjusted version of our method, specifically for genomic data enriched on the "1240k" positions (--capture). The only difference is the use of different assignment borders based on the number of SNPs per chromosome present on the "1240k" capture array commonly used in ancient DNA studies. The "autosomal baseline" N_a was defined as the sum of SNPs on chromosomes 1 through 22, N_a = 1,058,144 (excluding 13, 18, 21 as above). The mean R_x for XX individuals using target enrichment data was calculated as the number of SNPs on chromosome X (N_x = 49,704) divided by N_a (meanR_x for XX = 0.047). The mean R_x for XY individuals was (meanR_x for XX) / 2, i.e. meanR_x for XY = 0.023. Thus, the classification thresholds were defined at the mid-point between those two estimates and the upper R_x limit for XX was the mid-point between meanR_x for two X and meanR_x for three X chromosomes (meanR_x for XXX = 0.070). Regarding chromosome Y, the number of SNPs on the array (N_y = 32,670) divided by N_a equals to meanR_y for XY = 0.03. However, empirical R_y estimates from published data[83] had much smaller values than this theoretical R_y estimate, possibly due to the abundance of repeat regions on the Y chromosome that complicate sequence alignment[78]. Since the complete absence of chromosome Y results in R_y values close to zero, we decided to define the lower R_y limit for XY individuals to a value closer to those observed in real data (lowerR_y for XY = 0.004). Our method identified the 2 reported aneuploidy cases from this study too (ALM062 (47,XXX) and CLL011(47,XXY)), exhibiting robustness both in "shotgun" and "capture" sequenced genomes.

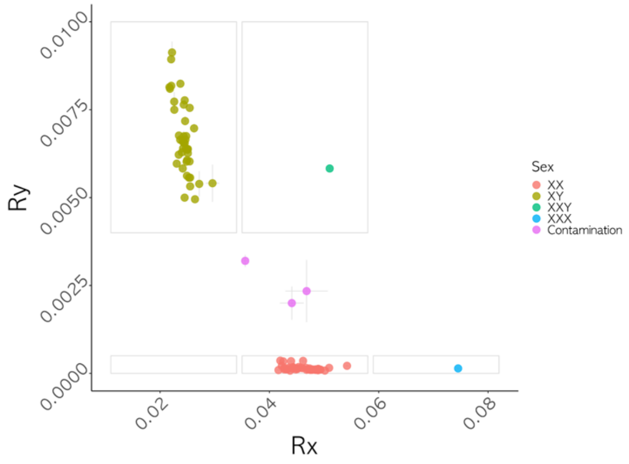

Supplementary figure 3: Application of our method using the --capture option (optimized for libraries enriched for the 1240k SNP array) on all individuals from Villalba-Mouco et al. 2021[83]. Our method identified the sex of XX and XY individuals, as well as contaminated libraries and two individuals with aneuploidy on sex chromosomes that were also reported in the original study ((ALM062 (47,XXX) and CLL011(47,XXY)).

Supplementary References

1. Knudtzon, J. & Svane, S. Turner's syndrome associated with chronic inflammatory bowel disease. A case report and review of the literature. *Acta Med. Scand.* **223**, 375–378 (1988).
2. Ostberg, J. & Conway, G. Screening for iron deficiency and anaemia in adults with Turner syndrome. *Endocr. Abstr.* **5**, (2003).
3. Stuart-Macadam, P. & Kent, S. *Diet, demography, and disease : changing perspectives on anemia.* (Aldine de Gruyter, 1992).
4. Arrieta, M., Ramos Gaspar, R. & Santos, A. L. Paleopathological diagnosis of a proportionate short stature on a female skeleton from the Coimbra collection: Turner syndrome versus other causes. *Int J Paleopathol* **33**, 234–244 (2021).
5. AlQahtani, S. J. Atlas of tooth development and eruption. *American Journal of Physical Anthropology* 54–54 (2009).
6. Trakinienė, G. *et al.* Genetic and environmental influences on third molar root mineralization. *Arch. Oral Biol.* **98**, 220–225 (2019).
7. Alhazmi, A. *et al.* Timing and rate of spheno-occipital synchondrosis closure and its relationship to puberty. *PLoS One* **12**, e0183305 (2017).
8. Coqueugniot, H. & Weaver, T. D. Brief communication: infracranial maturation in the skeletal collection from Coimbra, Portugal: new aging standards for epiphyseal union. *Am. J. Phys. Anthropol.* **134**, 424–437 (2007).
9. Schaefer, M., Scheuer, L. & Black, S. M. *Juvenile Osteology: A Laboratory and Field Manual.* (Academic, 2009).
10. Shirley, N. R. & Jantz, R. L. Spheno-occipital synchondrosis fusion in modern Americans. *J. Forensic Sci.* **56**, 580–585 (2011).

11. McDonald, S. W. & Miller, J. When does the spheno-occipital synchondrosis close? *Clin. Anat.* **35**, 512–525 (2022).

12. Doswell, B. H., Visootsak, J., Brady, A. N. & Graham, J. M., Jr. Turner syndrome: an update and review for the primary pediatrician. *Clin. Pediatr.* **45**, 301–313 (2006).

13. Huang, A. C., Olson, S. B. & Maslen, C. L. A Review of Recent Developments in Turner Syndrome Research. *J Cardiovasc Dev Dis* **8**, (2021).

14. Ranke, M. B. & Saenger, P. Turner's syndrome. *Lancet* **358**, 309–314 (2001).

15. Gravholt, C. H. *et al.* Marked disproportionality in bone size and mineral, and distinct abnormalities in bone markers and calcitropic hormones in adult turner syndrome: a cross-sectional study. *J. Clin. Endocrinol. Metab.* **87**, 2798–2808 (2002).

16. Outram, A. K. Bone fracture and within-bone nutrients: An experimentally based method for investigating levels of marrow extraction. in *Consuming Passions and Patterns of Consumption* (ed. Milner, P. M. A.) 51–63 (Cambridge: McDonald Institute for Archaeology, 2002).

17. Wheatley, B. P. Perimortem or postmortem bone fractures? An experimental study of fracture patterns in deer femora. *J. Forensic Sci.* **53**, 69–72 (2008).

18. Rinaldo, N., Zedda, N., Bramanti, B., Rosa, I. & Gualdi-Russo, E. How reliable is the assessment of Porotic Hyperostosis and Cribra Orbitalia in skeletal human remains? A methodological approach for quantitative verification by means of a new evaluation form. *Archaeol. Anthropol. Sci.* **7**, 3549–3559 (2019).

19. Brock, F., Higham, T., Ditchfield, P. & Ramsey, C. B. Current Pretreatment Methods for AMS Radiocarbon Dating at the Oxford Radiocarbon Accelerator Unit (Orau). *Radiocarbon* **52**, 103–112 (2010).

20. DeNiro, M. J. Postmortem preservation and alteration of in vivo bone collagen isotope ratios in relation to palaeodietary reconstruction. *Nature* **317**, 806–809 (1985).

21. van Klinken, G. J. Bone Collagen Quality Indicators for Palaeodietary and Radiocarbon Measurements. *J. Archaeol. Sci.* **26**, 687–695 (1999).

22. Ramsey, C. B. Development of the Radiocarbon Calibration Program. *Radiocarbon* **43**, 355–363 (2001).

23. Reimer, P. J. *et al.* The IntCal20 Northern Hemisphere Radiocarbon Age Calibration Curve (0–55 cal kBP). *Radiocarbon* **62**, 725–757 (2020).

24. Moorrees, C. F., Fanning, E. A. & Hunt, E. E., Jr. AGE VARIATION OF FORMATION STAGES FOR TEN PERMANENT TEETH. *J. Dent. Res.* **42**, 1490–1502 (1963).

25. Scheuer, L. & Black, S. *Developmental Juvenile Osteology*. (Elsevier Academic Press, 2000).

26. Smith, B. H. Standards of human tooth formation and dental age assessment. in *Advances in dental anthropology* (ed. Kelley, M.A. and Larsen, C.S) 143–168 (Wiley-Liss Inc., 1991).

27. Elamin, F. & Liversidge, H. M. Malnutrition has no effect on the timing of human tooth formation. *PLoS One* **8**, e72274 (2013).

28. AlQahtani, S. J., Hector, M. P. & Liversidge, H. M. Accuracy of dental age estimation charts: Schour and Massler, Ubelaker and the London Atlas. *Am. J. Phys. Anthropol.* **154**, 70–78 (2014).

29. Phenice, T. W. A newly developed visual method of sexing the os pubis. *Am. J. Phys. Anthropol.* **30**, 297–301 (1969).

30. Sutherland, L. D. & Suchey, J. M. Use of the ventral arc in pubic sex determination. *J. Forensic Sci.* **36**, 501–511 (1991).

31. Ubelaker, D. H. & Buikstra, J. E. *Standards for Data Collection from Human Skeletal Remains: Proceedings of a Seminar at the Field Museum of natural history*. (Arkansas Archeological Survey, 1994).
32. Mays, S. *The archaeology of human bones (2nd edition)*. (Routledge, 2010).
33. Walker, P. L. Sexing skulls using discriminant function analysis of visually assessed traits. *Am. J. Phys. Anthropol.* **136**, 39–50 (2008).
34. D. Gentry Steele and Claud A. Bramblett. *The anatomy and biology of the human skeleton.* (Texas A&M University Press, 1988).
35. Brickley, M. & McKinley, J. Determination of sex from archaeological skeletal material. in *Guidance to standards for recording human skeletal remains* (2004).
36. Teague, S, and Ford, B, et al. Between Oxford and the River Cherwell - Anglo-Saxon, Medieval and Post-medieval evidence from Longwall Quad and Library, Magdalen College. in *Oxford: Archaeological excavations* 2011-14 (Oxford Archaeology).
37. Gilchrist, R. & Sloane, B. Requiem: the Medieval Monastic Cemetery in Britain. (2005).
38. McKinley, J. I. Compiling a skeletal inventory: disarticulated and co-mingled remains. in *Guidance to standards for recording human skeletal remains* (ed. McKinley, M. B. A. J.) 14–17 (BABAO and IFA, 2004).
39. Ferembach, D, Schwidetzky, I, and Stoukal, M. Recommendation for Age and Sex Diagnoses of skeletons. J. *Hum. Evol.* **9**, 517–549 (1980).
40. Schwartz, J. H. *Skeleton keys: An introduction to human skeletal morphology, development and analysis, 2nd edition.* (Oxford University Press, 1995).
41. Bass, W. M. *Human osteology : a laboratory and field manual.* (Missouri Archaeological Society, 1987).

42. Brooks, S. & Suchey, J. M. Skeletal age determination based on the os pubis: A comparison of the Acsádi-Nemeskéri and Suchey-Brooks methods. *Hum. Evol.* **5**, 227–238 (1990).
43. Lovejoy, C. O., Meindl, R. S., Pryzbeck, T. R. & Mensforth, R. P. Chronological metamorphosis of the auricular surface of the ilium: a new method for the determination of adult skeletal age at death. *Am. J. Phys. Anthropol.* **68**, 15–28 (1985).
44. Buckberry, J. L. & Chamberlain, A. T. Age estimation from the auricular surface of the ilium: a revised method. *Am. J. Phys. Anthropol.* **119**, 231–239 (2002).
45. Brothwell, D. R. *Digging up bones. Third edition, revised and updated.* vol. 59 (Cornell University Press, 1981).
46. Miles, A. E. Assessment of the Ages of a Population of Anglo-Saxons from Their Dentitions. *Proc. R. Soc. Med.* **55**, 881–886 (1962).
47. Trotter, M. Estimation of stature from intact long bones. in *Personal identification in mass disasters* (ed. Stewart, T. D.) 71–83 (Smithsonian Institution Press, 1970).
48. Smyth, C. M. & Bremner, W. J. Klinefelter syndrome. *Arch. Intern. Med.* **158**, 1309–1314 (1998).
49. Kanakis, G. A. & Nieschlag, E. Klinefelter syndrome: more than hypogonadism. *Metabolism* **86**, 135–144 (2018).
50. Hillson, S. *Dental Anthropology*. (Cambridge University Press, 1996).
51. Roberts, C. A. & Manchester, K. *The Archaeology of Disease*. (Cornell University Press, 2007).
52. Primeau, C., Arge, S. O., Boyer, C. & Lynnerup, N. A test of inter- and intra-observer error for an atlas method of combined histological data for the evaluation of enamel hypoplasia. *J. Archaeol. Sci. Rep.* **2**, 384–388 (2015).

53. Dunbar, E., Cook, G. T., Naysmith, P., Tripney, B. G. & Xu, S. AMS 14C Dating at the Scottish Universities Environmental Research Centre (SUERC) Radiocarbon Dating Laboratory. *Radiocarbon* **58**, 9–23 (2016).

54. Pearson, G. W. & Stuiver, M. High-Precision Calibration of the Radiocarbon Time Scale, 500–2500 bc. *Radiocarbon* **28**, 839–862 (1986).

55. Arneborg, J. *et al.* Change of Diet of the Greenland Vikings Determined from Stable Carbon Isotope Analysis and 14C Dating of Their Bones. *Radiocarbon* **41**, 157–168 (1999).

56. Reimer, P. J. *et al.* IntCal13 and Marine13 Radiocarbon Age Calibration Curves 0–50,000 Years cal BP. *Radiocarbon* **55**, 1869–1887 (2013).

57. Loe, L. & Rowland, S. A63 *Castle Street Improvement, Trinity Burial Ground, Kingston upon Hull, East Yorkshire. Oxford Archaeology unpublished interim document and updated project design for analysis, dissemination, and archiving*. (Oxford Archaeology, 2022).

58. Berry, A. C. & Berry, R. J. Epigenetic variation in the human cranium. *Journal of Anatomy* **101**, 361–379 (1967).

59. Barnes, E. *Atlas of Developmental Field Anomalies of the Human Skeleton: A Paleopathology Perspective*. (Wiley, 2012).

60. Djuric, M. *et al.* Porotic lesions in immature skeletons from Stara Torina, late medieval Serbia. *Int. J. Osteoarchaeol.* **18**, 458–475 (2008).

61. Mangas-Carrasco, E. & López-Costas, O. Porotic hyperostosis, cribra orbitalia, femoralis and humeralis in Medieval NW Spain. *Archaeol. Anthropol. Sci.* **13**, (2021).

62. Rothschild, B. M., Zdilla, M. J., Jellema, L. M. & Lambert, H. W. Cribra orbitalia is a vascular phenomenon unrelated to marrow hyperplasia or anemia: Paradigm shift for cribra orbitalia. *Anat. Rec.* **304**, 1709–1716 (2021).

63. Roberts, C. A. A bioarcheological study of maxillary sinusitis. *Am. J. Phys. Anthropol.* **133**, 792–807 (2007).
64. Cox, M. & Mays, S. *Human Osteology: In Archaeology and Forensic Science*. (Cambridge University Press, 2000).
65. Roberts, C. A. & Cox, M. *Health and disease in Britain: from prehistory to the present day*. (Sutton Publishing, 2003).
66. Finnegan, M. Non-metric variation of the infracranial skeleton. J. *Anat.* **125**, 23–37 (1978).
67. Lewis, M. E. *The Bioarchaeology of Children: Perspectives from Biological and Forensic Anthropology*. (Cambridge University Press, 2007).
68. Knüsel, C. Activity-related change. in *Blood Red Roses: The Archaeology of a Mass Grave from the Battle of Towton AD 1461* (ed. Fiorato, V., Boylston, A. And Knüsel, C.) 103–118 (Oxbow books, 2000).
69. Stirland, A. Human remains. in *Before the Mast: Life and Death Aboard the Mary Rose. The Archaeology of the Mary Rose*: 4 (ed. Allen, J. G. A. M.) 516–544 (Oxbow Books, 2005).
70. Mann, R. W. and Hunt, D. R. *Photographic Regional Atlas of Bone Disease: A Guide to Pathologic and Normal Variation in the Human Skeleton*. (Charles C. Thomas Publisher, 2005).
71. Dandy, D. J. and Edwards, D. J. *Essential Orthopaedics and Trauma*. (Churchill Livingstone, 2003).
72. Skoglund, P. *et al.* Separating endogenous ancient DNA from modern day contamination in a Siberian Neandertal. *Proc. Natl. Acad. Sci. U. S. A.* **111**, 2229–2234 (2014).
73. 1000 Genomes Project Consortium *et al.* A global reference for human genetic variation. *Nature* **526**, 68–74 (2015).

74. Antonio, M. L. *et al.* Ancient Rome: A genetic crossroads of Europe and the Mediterranean. *Science* **366**, 708–714 (2019).

75. Margaryan, A. *et al.* Population genomics of the Viking world. *Nature* **585**, 390–396 (2020).

76. Bycroft, C. *et al.* The UK Biobank resource with deep phenotyping and genomic data. *Nature* **562**, 203–209 (2018).

77. Sun, H., Ding, J., Piednoël, M. & Schneeberger, K. findGSE: estimating genome size variation within human and Arabidopsis using k-mer frequencies. *Bioinformatics* **34**, 550–557 (2018).

78. Bachtrog, D. & Charlesworth, B. Towards a complete sequence of the human Y chromosome. *Genome Biol.* **2**, REVIEWS1016 (2001).

79. Subramanian, S., Mishra, R. K. & Singh, L. Genome-wide analysis of microsatellite repeats in humans: their abundance and density in specific genomic regions. *Genome Biol.* **4**, R13 (2003).

80. Freeman, J. L. *et al.* Copy number variation: new insights in genome diversity. *Genome Res.* **16**, 949–961 (2006).

81. Hallast, P. *et al.* Assembly of 43 human Y chromosomes reveals extensive complexity and variation. *Nature* (2023) doi:10.1038/s41586-023-06425-6.

82. Zhao, Y. *et al.* Detection and characterization of male sex chromosome abnormalities in the UK Biobank study. *Genet. Med.* **24**, 1909–1919 (2022).

83. Villalba-Mouco, V. *et al.* Genomic transformation and social organization during the Copper Age-Bronze Age transition in southern Iberia. *Sci Adv* **7**, eabi7038 (2021).
