# Supplementary material for: Detection of chromosomal aneuploidy in ancient genomes
Source: Commun Biol. 2024 Jan 11;7:14. doi: 10.1038/s42003-023-05642-z (PMC10784527; doi:10.1038/s42003-023-05642-z)
Supplement: Supplementary file 2 — Description of additional supplementary files [file 42003_2023_5642_MOESM2_ESM.docx]

Description of Additional Supplementary Files

**File name:** Supplementary Data 1

**Description:** Sequencing metrics (number of sequences, percentage of human sequences, percentage of deaminated ancient DNA sequences, nuclear and mitochondrial coverage, uniparental haplogroups, contamination estimates, karyotypes) for the 6 individuals whose genomic data are first published in this study.

**File name:** Supplementary Data 2

**Description:** Source data for Figure 1a and Supplementary Figure 2.

**File name:** Supplementary Data 3

**Description:** Source data for Figure 3.

**File name:** Supplementary Data 4

**Description:** Source data for Figure 2b-c.
